# Treatment of hepatic metastases from medullary thyroid cancer with transarterial embolisation

**DOI:** 10.1186/s41747-017-0013-6

**Published:** 2017-06-29

**Authors:** Peter Hughes, Nuala A. Healy, Cliona Grant, J. Mark Ryan

**Affiliations:** 1 Department of Radiology, St. James’s Hospital, Dublin, Ireland; 2 Department of Oncology, St. James Hospital, Dublin, Ireland

**Keywords:** Medullary thyroid cancer, Metastases, Transarterial embolisation, Interventional radiology, Serum calcitonin

## Abstract

Transarterial chemoembolisation (TACE) is well established in the treatment of primary hepatocellular carcinoma and of metastatic disease from colorectal and neuroendocrine tumours. There are few published studies on the effectiveness of treating hepatic metastases from medullary thyroid carcinoma with chemoembolisation and none to our knowledge utilising bland particle transarterial embolisation (TAE).

Here we describe the management of multifocal hepatic metastases from medullary thyroid cancer in a 39-year-old woman who underwent bland particle TAE with a biochemical and radiological response and discuss the potential for a wider scope of clinical application for bland TAE in hepatic metastases.

## Key points

- Prior reports have evaluated TACE in metastatic medullary thyroid cancer.
- Bland particle TAE is a potentially useful treatment for metastatic medullary thyroid cancer.
- Serum calcitonin is useful in evaluating the response to treatment.

## Introduction

Medullary thyroid cancer (MTC) is a well differentiated neuroendocrine tumour of the parafollicular or C cells of the thyroid and accounts for 3–5% of all thyroid cancers [1]. Distant metastases are present at diagnosis in up to 17% of patients and discovered on subsequent surveillance in up to 38% of patients [1]. Metastases may be associated with tumour hormone production causing symptoms such as diarrhoea, flushes or even Cushing’s syndrome. Metastatic MTC is considered incurable with a five-year survival rate of 28% and treatment is aimed at controlling the symptoms of hormonal excess.

There are only a few published reports of the treatment of metastatic MTC with transarterial chemoembolisation (TACE) and none to our knowledge with transarterial embolisation (TAE) [2–4]. There remains some controversy about the superiority of TACE over TAE. There is currently no definitive evidence to support an additional benefit arising from adding a chemotherapeutic agent, while there is evidence to suggest that the addition of a chemotherapeutic agent can contribute to hepatic and systemic toxicity [5, 6]. There is currently no specific chemotherapeutic agent that can be bound to an embolic particle that has been shown to have a significant chemotherapeutic effect in metastatic MTC [7]. In this context, we aimed to determine if TAE might be a viable treatment option in metastatic MTC.

It is important to note that approximately 25% of MTC is related to a mutation in the RET proto-oncogene. If considering hepatic embolisation, it is crucial to exclude hepatic metastases from a pheochromocytoma in patients with a background of multiple endocrine neoplasia type 2. Unwitting embolisation of pheochromocytoma metastases has been resulted in death secondary to an ensuing hypertensive crisis [8].

## Case illustration

**Fig. 1:**
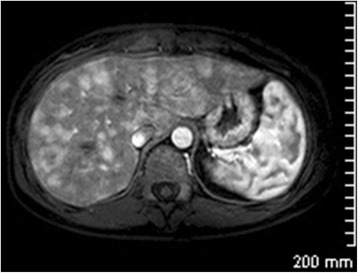
Pre-treatment axial T1-weighted contrast-enhanced MRI showing innumerable avidly enhancing, bilobar hepatic metastases

A 39-year-old woman presented with a left neck swelling which underwent sonographic evaluation and fine needle aspiration. Cytology demonstrated medullary thyroid carcinoma with positive immunostaining for calcitonin and carcinoembryonic antigen in an adjacent lymph node confirming local spread. Baseline positron emission tomography/computed tomography (PET/CT) and magnetic resonance imaging (MRI) revealed innumerable metastases within both hepatic lobes (Fig. 1). Serum calcitonin was > 2000 pg/ml (normal range < 11.5 pg/ml). Following referral to cancer genetics, germline deoxyribose nucleic acid analysis yielded no mutation in the REarranged during Transfection (RET) proto-oncogene.

The patient was treated with vandetanib to downstage the tumour prior to resection. Vandetanib is an oral small-molecule tyrosine kinase inhibitor that targets vascular endothelial growth factor receptors 2 and 3, RET, and, at higher concentrations, epithelial growth factor receptor. It has been shown to improve progression-free survival and tumour response in patients with unresectable, locally advanced or metastatic disease [9]. Three months later, serum calcitonin nadired at 353 pg/ml. Restaging PET/CT and MRI confirmed some reduction in the volume of metastases but showed significant residual burden of hepatic disease.

Vandetanib was held in anticipation of surgical resection of the primary tumour and within four weeks the serum calcitonin had risen to to 663 pg/ml. Six weeks following Vandetanib cessation, the patient underwent total thyroidectomy and bilateral central neck dissection with a left modified radical neck dissection. The resection specimen revealed a 3-cm tumour with extra thyroidal extension and lymphovascular invasion, with a total 14 out of 43 nodes positive for tumour involvement (American Joint Committee on Cancer staging pT3 pNb1).

On serial monitoring after thyroidectomy, calcitonin steadily increased from 3,131 pg/ml on the fifth postoperative day to 17,764 pg/ml 100 days later, a doubling time of six weeks.

The patient remained asymptomatic and was reticent to restart systemic therapy in consideration of its associated toxicities. However, given the significant rise in tumour markers, a decision was taken to perform embolisation of the hepatic metastases.

TAE was performed in two stages: first the right hepatic lobe followed by the left hepatic lobe eight days later. Both procedures were carried out through 5Fr right femoral access. Using a Mikaelsson catheter, selective catheterisation was performed of the coeliac and superior mesenteric arteries and arteriography performed. The proper hepatic artery was selectively catheterised using a microcatheter and selective arteriography was performed.

**Fig. 2:**
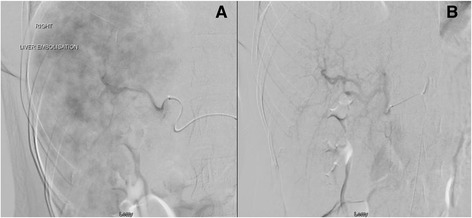
Digital subtraction angiography before and after embolisation of the right hepatic artery. The pre-embolisation image (**a**) demonstrates multiple hypervascular liver metastases, while the volume of tumour blush is significantly reduced on the post-embolisation image (**b**)

During the first procedure, selective right hepatic arteriography revealed a significant disease burden within the right lobe of the liver. The right hepatic artery was embolised with 100-μm microspheres (Embosphere, Merit Medical, Utah, USA). During the second procedure, repeat right hepatic arteriography revealed a small volume of residual disease; therefore, further subselective right hepatic artery embolisation using 100-μm microspheres was performed. This was followed by selective embolisation of the left hepatic artery and of a large accessory left hepatic artery arising from the left gastric artery (Fig. 2). The patient developed mild post-embolisation cholecystitis after the second procedure, settled with conservative methods. She was discharged home well six days later.

**Fig. 3:**
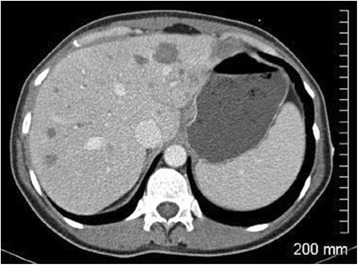
Contrast-enhanced axial CT image one day after TAE illustrating numerous hypoattenuating foci consistent with devascularised metastases

**Fig. 4:**
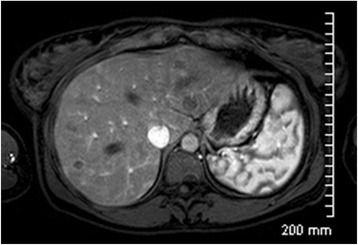
T1-weighted contrast-enhanced axial MR image four weeks after TAE showing a reduction in the volume of enhancing hepatic metastases

While the pre-treatment metastases were relatively occult on CT, a CT one day following the second embolisation procedure showed multiple hypoenhancing foci throughout both hepatic lobes consistent with devascularised metastases (Fig. 3). A follow-up liver MRI four weeks later showed significantly reduced enhancement in the multiple hepatic metastases consistent with radiological treatment response (Fig. 4). The serum calcitonin measured four weeks post procedure was 6,065 pg/mL.

## Discussion

Prior studies have described treatment of MTC hepatic metastases with TACE utilising Lipiodol and doxorubicin in a similar manner to other forms of metastatic neuroendocrine tumour, albeit with a small number of patients [2–4]. Fromigué et al. demonstrated a partial radiological tumour response in 42% (n = 5) of patients up to 17 months after TACE and stabilisation of disease on imaging in 42% (n = 5) of patients up to 24 months after chemoembolisation [3]. Lorenz et al. demonstrated a biochemical and radiologic response in 50% of patients (n = 5), with a biochemical response defined as a 10% fall in tumour markers [4].

Serum calcitonin is a highly sensitive and specific tumour marker for MTC [10]. At least four sequential levels over time are required to provide a measurement of doubling time, which closely parallels tumour volume and is therefore a strong predictor of progression and survival. A doubling time of less than six months has been shown to be associated with a 75% risk of cancer-related death in the following five years [10].

In the current case, the doubling time was six weeks, placing the patient in a high-risk category for further disease progression. The serum calcitonin levels had decreased by 65.9% to 6,065 pg/ml at four weeks after TAE, a finding consistent with a clinically relevant reduction in the volume of viable tumour. Post-treatment liver MRI also demonstrated decreased enhancement of the hepatic metastases.

While there is at least a conceptual logic for adding a chemotherapeutic agent to which the tumour is sensitive, there is currently no specific chemotherapeutic agent that can be bound to an embolic particle that has been shown to have a significant chemotherapeutic effect in metastatic MTC. The purported benefit of drug-eluting beads is based on a low peak plasma concentration of the chemotherapeutic drugs resulting in exposure of the tumour to the therapeutic agents with less exposure of healthy liver tissue [11]. Even allowing for this, the addition of doxorubicin to embolic agents has been shown to be associated with an increase in arterial and parenchymal necrosis and the establishment of an inflammatory response resulting in disturbances in liver metabolism [12].

It is also debatable whether the addition of a chemotherapeutic agent provides any additional benefit over bland particle TAE alone. It remains unclear whether the treatment effect seen is primarily the result of the ischaemic effect of the embolic agent and to what extent, if any, the addition of chemotherapeutic agent contributes to this effect. Bland TAE has previously been described as effective in the management of liver metastases from multiple tumour types, including metastatic sarcoma and gastrointestinal stromal tumour [13, 14]. Bonomo et al. reported a benefit from selective TAE using bland particles to achieve a maximal ischaemic effect, arguing that ischaemia is the dominant influence on the outcome [15]. A recent randomised controlled trial comparing bland TAE with TACE in 101 patients in the treatment of hepatocellular carcinoma found there was no apparent difference between the two treatment groups [5].

Given this uncertainty, and taking into account the added cost and potential side effects associated with TACE compared with TAE, we propose that TAE may be an effective treatment in cases of metastatic MTC.

To summarise, while a small number of previous studies have shown promise in treating metastatic MTC with TACE, this case demonstrates that bland particle embolisation alone may achieve a similar radiological and biochemical response. Serum calcitonin may be useful as a tumour marker in evaluating the response to treatment, although how this relates to progression-free survival needs to be further elucidated. We believe that TAE may be considered as a potentially useful adjunctive therapy in achieving stabilisation of disease in patients with hepatic metastases from MTC.

## Abbreviations

MRI: Magnetic resonance imaging
MTC: Medullary thyroid cancer
PET/CT: Positron emission tomography/computed tomography
RET: Rearranged during transfection
TACE: Transarterial chemoembolisation
TAE: Transarterial embolisation
